# Children and Adolescents' Mental Health Following COVID-19: The Possible Role of Difficulty in Emotional Regulation

**DOI:** 10.3389/fpsyt.2022.865435

**Published:** 2022-06-20

**Authors:** Meirav Hen, Vered Shenaar-Golan, Uri Yatzker

**Affiliations:** ^1^Psychology Department, Tel-Hai Academic College, Tel Hai, Israel; ^2^Social Work Department, Tel-Hai Academic College, Tel Hai, Israel; ^3^Ziv Medical Center, Safed, Israel

**Keywords:** COVID-19, difficulties in emotion regulation, gender, children, mental symptoms

## Abstract

The COVID-19 pandemic has shattered routines throughout the world, creating closures and social isolation. Preliminary studies conducted during the pandemic have shown that children and adolescents are mainly affected by social distancing and the lack of a supportive framework. The purpose of the present study was to compare mental health symptoms of 430 children and adolescents who sought mental health services in the community before vs. during the pandemic. The study examined children's perceived burden of the pandemic, reports of emotional and behavioral problems (SDQ) anxiety (SCARED), depressed moods (SMFQ-C), and difficulty in emotional regulation (DERS), as well as intervening variables such as age and gender. Furthermore, the effect of difficulty in emotional regulation on children's mental health symptoms was explored. Findings indicate an increase in all mental health symptoms excluding anxiety, during the pandemic. Boys reported more difficulty in emotional regulation during the pandemic than before, and girls reported more emotional and behavioral problems. Children reported an increase in emotional and behavioral problems and adolescents in peer relationship problems. Difficulty in emotional regulation predicted all mental health symptoms in both samples, more so in girls and adolescents. These initial findings support the need for further studies to examine the long-term mental health effects of COVID-19 pandemic on children and adolescents.

## Introduction

In March 2020, the World Health Organization declared a general state of emergency following the outbreak of the coronavirus COVID-19 (1). The pandemic brought with it an immediate and complete collapse of all life routines, social isolation, economic uncertainty, and existential anxiety from the disease itself (2). For many people this apocalyptic situation was a serious burden and required them to mobilize exceptional mental resources to cope and adapt to the new reality (3). For instance, in a series of studies Brailovskaia et al. (4, 5) surveyed adults from Germany, Italy, Russia and Spain and found that burden by COVID-19 was significantly and positively associated with depression symptoms, and negatively associated with physical activity. Their findings also revealed that social media use is positively associated with stress symptoms and experienced burden and that stress symptoms mediated the link between social media use and burden. Positive associations were between burden by COVID-19 and addictive social media use, mediated by the level of perceived sense of control (4–6). Further studies that examined adults' mental health following the pandemic revealed an increase in loneliness and symptoms of depression mostly in females (7), anxiety and circadian rhythm dysregulation (8), stress, suicide, and substance use mostly in men (9).

Although children and adolescents often represent a milder course of COVID-19 compared to adults, the measures taken to contain the disease, such as school closures and social isolation, have negatively impacted the mental health and well-being of this young population (10). Long hours online, distance learning, inactivity, lack of physical activity, boredom, lack of sleep and changes in eating habits have impaired the self-image of children and adolescents, their ability to engage in the normal, age-appropriate developmental tasks, and increased their negative moods (11, 12). Children and adolescents have also been impacted by the marital and family dynamics of stress, neglect and violence following the pandemic, and the concern for the wellbeing of parents and other family members (13, 14). Most preliminary studies of the pandemic indicated an increase in stress, anxiety, post-traumatic symptoms and depressed moods, and a decrease in health-related quality of life in children and adolescents around the world, including in Israel (10, 15–17).

For some children the feelings of stress and pressure created by the pandemic connected to a mental state that was already precarious and exacerbated it even more (18). It is important to note that in the first year of the COVID-19 pandemic there was a marked decrease in the number of visits to health clinics, and children who had previously been under continuous treatment were less likely to seek treatment even if their condition worsened (19). Part of this is related to the general closure and the fear of possible infection in populated places such as health and mental health centers. However, it seems that the closure of educational institutions and the subsequent lack of monitoring of children's development through education, especially in older youth, was also a significant factor in the decrease in visits to health and mental health clinics (20).

It was further found that the pandemic's continuing duration not only exacerbated its negative ripple effect but also intensified and deepened the difficulty for certain populations. For example, Zhou et al. (21) found that girls, more than boys, suffered from symptoms of stress and anxiety immediately upon the onset of the epidemic and later, from symptoms of depression. Another study found that adolescents 14 years of age and older, more than children up to 14 years old, showed signs of depression, and that boys showed more anger and problematic behavior (22). Furthermore, continued parental pressure during the pandemic, more “online” hours, background illnesses, low socioeconomic status and abusive relationships at home were all revealed as significant risk factors for children's and adolescents' mental wellbeing, while social relationships, a supportive community and relationships with parents were positive factors for children and adolescents (22, 23).

Achterberg et al. (24), in a longitudinal study among children, age 10–13, found that children's externalizing behavior changes before vs. during the pandemic were mediated by perceived stress. Other studies conducted in Italy and Spain found an increase in extroverted behavioral problems especially among young boys (12, 25, 26), and a study in the UK showed a 10% increase in the general level of emotional problems, a 20% increase in symptoms of inattention and hyperactivity, and a 30% increase in the level of behavioral problems in children. That study found that adolescents had only a moderate increase in behavioral problems and inattention and a decrease in the general level of abnormality (27). In contrast, another study showed a significant increase in the level of depression in young children but found no significant change in their levels of anxiety and emotional and behavioral symptoms during the pandemic (28).

In this context, Breaux et al. (29) contended that most studies conducted during the pandemic on adolescents were based on online surveys in the “normal” or “healthy” population and did not provide a sufficiently accurate picture of the clinical population treated in mental health centers. According to the authors and to other studies, the condition of the clinical populations became much more acute, and those who were considered “borderline” before the pandemic and were able to function in educational settings at least, were now experiencing even more difficulty (29, 30).

For example (29), studied adolescents suffering from ADHD during the pandemic, and argued that they are at high risk for developing emotional and behavioral disorders during the pandemic due to their stage of developmental, their difficulties in emotional regulation, and their need of a framework and especially of a peer group. The combination of difficulty in emotional regulation and increased anxiety and depression levels in the early months of the COVID-19 pandemic was found to compound the likelihood of intensified emotional and behavioral problems experienced by children and adolescents (31, 32). Previous studies examining the impact of stressful situation have shown that emotion regulation plays an important role in determining the effects on an individual [e.g., (33, 34)]. Some early evidence among adults show that emotion regulation abilities are linked to better mental health functioning during the COVID-19 pandemic, suggesting that having well-developed emotion regulation abilities may serve as a protective factor (35, 36).

Emotion regulation (ER) is often defined as the ability to monitor, evaluate, and modify emotions that arise following a stimulus, and respond to them adaptively (37). This ability refers to a set of processes that people use to modulate, consciously or not, their emotional states to respond to environmental requests in an adaptive way (38, 39). An emotion regulation perspective has been widely adopted in the study of internalizing disorders such as depression or anxiety disorders, suggesting that an inability to regulate negative emotions is a central feature of depression and anxiety (40). Difficulties in emotion regulation may arise due to the inability to recognize, understand, or accept certain emotional states, thereby they are recognized as a risk factor for behavioral (41) and emotional problems such as anxiety and depression (39). Difficulties in emotional regulation have been consistently described as a transdiagnostic vulnerability factor that is linked to numerous difficulties and forms of psychopathology across the lifespan (42, 43). This is particularly true for adolescents who are in a stage of development- marked by overwhelming emotion reactivity, impulsive behavior and neurobiological processes implicated in ER (44).

For example, studies show that five-year-old children are already able to identify emotions and choose how to express them according to the situation in which they find themselves (45). On the other hand, when children are impulsive or find themselves in an unregulated environment it will be much more difficult for them to develop their emotional self-regulatory capacity (46). It is commonly thought that the emotional regulation ability usually matures toward early adulthood, and earlier in females—during adolescence (47).

Late childhood and adolescence are developmental periods characterized by stress and emotional turmoil, due to physical development and hormonal rush, as well as difficulty in emotional regulation (48). The combination of a turbulent emotional world and a developing system of emotional regulation creates inadequacy and a heightened level of perception and increased responsiveness. This often leads to mental distress manifested in internalized and externalized symptoms that may be amplified when the internal turmoil typical of adolescence meets an extreme external situation such as COVID-19 (49).

### Aim of the Current Research

Taking all this into consideration and based on the scientific literature reporting adverse changes in the mental state of children and adolescents in the world during the COVID-19 pandemic, as well as the paucity of studies based on children's own reporting and clinical samples of children and adolescents, the present study examines the current pandemic's burden and ripple effect on the mental health of children and youth who reach out to mental health clinics in Israel. The study compared anxiety, depressed moods, difficulty in emotional regulation and emotional and behavioral problems in children and adolescents who sought assistance from a mental health clinic before vs. during the COVID-19 pandemic. The study also tested the hypothesis that difficulty in emotional regulation constitute a cross-diagnostic variable that can explain mental struggles among children and youth who seek help during and regardless of the COVID-19 pandemic.

Study hypotheses:

1. There will be an increase in symptoms of anxiety and depressed moods, difficulty in emotional regulation and emotional and behavioral problems in children and adolescents during the current pandemic.
2. There will be an increase in the reporting of the abnormal range of emotional and behavioral problems during the COVID-19 pandemic.
3. Difficulty in emotional regulation will predict emotional and behavioral problems, anxiety and depressed moods in children and youth who were referred to a mental health clinic before vs. during the COVID-19 pandemic.

## Methods

### Participants

The sample included 430 children and adolescents who were referred to an ambulatory psychiatric clinic in a public hospital that serves a regional population. The sample included 172 (40.0%) boys and 258 (60.0%) girls between the ages 8–17 (M = 13.8, SD = 2.6). Two hundred and ninety-eight children and adolescents were referred to the clinic before the start of the COVID-19 pandemic (up to January 2020) and 132 during the pandemic (March–June 2021). The clinic provides evaluation and treatment for a variety of mental health problems. Children referred to the clinic are briefly assessed by a clinician and referred to a specific clinic for further evaluation and treatment. The children and adolescents who agreed to participate in this study completed a consent form and a self-report questionnaire about their mental health condition. All parents also provided informed consent for the present study. The study was approved by the IRB (Helsinki Committee ZIV0083-19) of Ziv Medical Center, Safed, Israel.

### Measures

For sociodemographic information, the child and adolescent survey included questions on age and gender and perceived burden of the pandemic, for the “during pandemic” sample only. The Strengths and Difficulties Questionnaire (SDQ) (50). The SDQ is a widely used screening tool for behavioral and emotional disorders in children and adolescents (51), with good convergent validity with clinician-rated diagnoses (52). The 25-item measure includes 5 subscales that assess internalizing (emotional problems subscale) and externalizing (hyperactivity and conduct subscales) symptoms, a subscale measuring difficulties with peers, and a subscale assessing prosocial behavior. High scores on the four “difficulties” scales represent a high degree of difficulty; a high score on the prosocial scale represents a high degree of prosocial behavior. According to published cut-offs, the participants were categorized based on the sum scores into groups according to their mental health (abnormal, borderline, and normal) (10). In the present study we used only the four difficulties subscales and the total subscale. Internal consistency in the current sample was: α = 0.74 for total SDQ, α = 0.70 for Emotional problems, α = 0.70 for Hyperactivity, α = 0.60 for Social problems, and α = 0.60 for Conduct problems.

The Screen for Child Anxiety Related Disorders (SCARED) (53) includes nine items on symptoms of generalized anxiety (e.g., “I worry about what is going to happen in the future”) which are presented with a 3-point response scale (0 = “not true or hardly ever true” to 2 = “very true or often true”). These 9 items are gathered in a sum score with higher scores indicating more severe symptoms of generalized anxiety. Internal consistency in the current sample was α = 0.93.

The Difficulties in Emotion Regulation Scale (DERS) (54) is a 36-item self-report measure scored on a 5-point Likerttype scale. Respondents indicate the frequency with which statements such as “I am clear about my feelings” apply to themselves. From the primary article, “The DERS items were chosen to reflect difficulties within the following dimensions of emotion regulation: (a) awareness and understanding of emotions; (b) acceptance of emotions; (c) the ability to engage in goal-directed behavior and refrain from impulsive behavior when experiencing negative emotions; and (d) access to emotion regulation strategies perceived as effective.” Regarding reliability (44), the DERS had high internal consistency (α = 0.93). Internal consistency in the current sample for the DERS total score was α = 0.93. All the DERS subscales (computed from the six factors obtained in the factor analysis) also had adequate internal consistency, with Cronbach's α > 0.80 for each subscale.

The Short Mood and Feelings Questionnaire for children (SMFQ-C) (55) is a brief, easy-to-administer, self-report measure of childhood and adolescent depression, designed for the rapid evaluation of core depressive symptomatology or for use in epidemiological studies. There are two parallel versions of this 13-item scale: one for parental reports and one for child/adolescent reports (self-report version). Responses are rated on a 3- point scale (0 = not true, 1 = sometimes true, and 2 = true). The SMFQ-C takes ~3 to 5 min to complete and has been validated with clinical and non-clinical samples. A score of 12 or above is commonly considered an indication of clinically significant depression. The SMFQ-C was originally designed for use with children. Internal consistency in the current sample was α = 0.90.

### Data Analysis

To investigate the perceived burden of the pandemic, the pandemic-specific items were examined via descriptive statistics (frequencies, means and standard deviations). To evaluate differences in DERS, SCARED, SMFQ-C, and SDQ before vs. during the pandemic, cross-sectional data from the pre pandemic study and the pandemic study were obtained; and two different subsamples were created. Although data analysis revealed an abnormal distribution of the data, based on the large sample, the continues variables and the Central Limit Theorem (CLT) (56), independent group t-tests were performed to examine differences in all variables between samples, stratified by gender and age. Furthermore, the difference in prevalence of normal and abnormal reports on all SDQ subscales between samples was analyzed. Finally, a multivariate regression analysis was performed with all data (Method: Enter), to explore the effect of difficulty in emotional regulation on SCARED, SMFQ-C, and SDQ total. Effects were described as mean differences and Cohens d-effect size measures. All analyses were performed using SPSS version 26 (57).

## Results

To learn about participants' perceived burden of the pandemic COVID-19, 124 (Boys- *n* = 43; Girls- *n* = 81; Children-*n* = 38; Adolescents- *n* = 86) “during the pandemic” (March-June 2021), participants were asked to answer to what degree they felt their feelings changed during the pandemic. Most participants (boys 50.0%, girls 72.3%, children 42.55%, adolescents 74.7%; mean 64.6%) reported they felt worse during the pandemic. Others (boys 36.4%, girls 21.7%, children 42.5%, adolescents 19.5%; mean 26%) felt there was no change, and some reported that their feelings improved during the pandemic (boys 13.6%, girls 6.0%, children 15.0%, adolescents 5.7%; mean 8.7%). To examine the relationship between “during pandemic” participants' perceived burden of the pandemic and mental health variables (DERS, SCARED, SMFQ-C, SDQ-Total) we conducted a Pearson coefficient test (see Table 1).

**Table 1:** Perceived burden of COVID-19 and DERS, SCARED, SMFQ-C and SDQ-total stratified by gender and age.

	**All (*n =* 124)**	**Boys (*n =* 43)**	**Girls (*n =* 81)**	**Age 8–13 (*n =* 38)**	**Age 14–17 (*n =* 86)**
DERS	−0.266 (0.003)	−0.218 (0.159)	−0.277 (0.012)	−0.192 (0.247)	−0.278 (0.009)
SCARED	−0.352 (<0.001)	−0.371 (0.014)	−0.312 (0.005)	−0.287 (0.081)	−0.311 (0.004)
SMFQ-C	−0.067 (0.452)	−0.167 (0.280)	0.003 (0.979)	−0.064 (0.697)	−0.098 (0.367)
SDQ total	−0.292 (0.001)	−0.082 (0.615)	−0.390 (<0.001)	−0.200 (0.243)	−0.260 (0.018)

The perceived burden of all participants was significantly and negatively associated with SCARED (r = −0.352, *p* < 0.001) SDQ-t (r = −0.292, *p* = 0.001), and DERS (r = −0.266, *p* = 0.003), but not with SMFQ-C (r = −0.067, *p* = 0.452) suggesting that feeling worse during the pandemic was associated with an increase in anxiety, emotional and behavioral problems, and difficulty in emotional regulation. For boys perceived burden was significantly associated only with anxiety (r = −0.371, *p* = 0.014), for girls perceived burden was associated with anxiety (r = −0.312, *p* = 0.005), emotional and behavioral problems (r = −0.390, *p* < 0.001), and difficulty in emotional regulation (r = −0.277, *p* = 0.012). For adolescents perceived burden was associated with anxiety (r = −0.311, *p* = 0.004), emotional and behavioral problems (r = −0.260, *p* < 0.018), and difficulty in emotional regulation (r = −0.278, *p* = 0.009).

To compare mental health variables (DERS, SCARED, SMFQ-C, SDQ total) between samples (before vs. during COVID-19), an independent group *t*-test was performed (see Table 2).

**Table 2:** Mental health variables in children and adolescents before vs. during the COVID-19 pandemic.

	**Before (*n* = 290)**		**During (*n* = 130)**				
	**M**	**SD**	**M**	**SD**	**t _**(418)**_**	***p***	**Cohen's D**
DERS	2.83	0.81	3.05	0.76	−2.43	0.016	0.280
SCARED	34.65	16.86	34.21	16.44	−0.25	0.804	0.026
SMFQ-C	11.64	7.96	13.57	8.18	−2.28	0.023	0.239
SDQ total	16.73	5.71	18.28	6.26	−2.44	0.015	0.258

Prior to examining the specific differences in gender and age on all mental health variables between the samples (before vs. during COVID-19), gender and age differences in all participants were examined (see Table 3).

**Table 3:** Mental health variables in children and adolescents by gender and age.

	**Boy** **(*n =* 162)**	**Girl** **(*n =* 254)**			**Age 8–13 (*n =* 170)**	**Age 14–17 (*n =* 250)**		
	**M (SD)**	**M (SD)**	**t_**(418)**_**	***P***	**M (SD)**	**M (SD)**	**t_**(418)**_**	***p***
DERS	2.75 (0.72)	3.00 (0.83)	−3.19	0.002	2.73 (0.72)	3.01 (0.83)	−3.59	<0.001
SCARED	31.00 (16.87)	36.88 (16.22)	−3.60	<0.001	32.46 (16.84)	35.90 (16.52)	−2.08	0.038
SMFQ–C	10.04 (7.82)	13.70 (7.91)	−4.67	<0.001	9.64 (7.57)	13.98 (7.94)	−5.60	<0.001
SDQ total	17.04 (5.59)	17.39 (6.06)	−0.63	0.536	17.12 (5.67)	17.34 (6.01)	−0.38	0.705

As can be seen in Table 3 there is a significant difference between boys and girls in all mental health variables: DERS [t(418) = −3.19; *p* = 0.002], SMFQ-C [t(418) = −4.67; *p* < 0.001], SCARED [t(418) = −3.60; *p* < 0.001] but not in the SDQ total [t(418) = −0.63; *p* = 0.536]. A significant difference between children and adolescents in all mental health variables was found as well: DERS [t(418) = −3.59; *p* < 0.001], SMFQ-C [t(418) = −5.60; *p* < 0.001], SCARED [t(418) = −2.08; *p* = 0.038] except in the SDQ total [t(418) = −0.38; *p* = 0.705]. In all variables, girls reported more difficulties than boys and adolescents more than children. Cohen's D effect was weak (>0.28).

Independent group *t*-tests were conducted to examine differences between all mental health variables among sample groups (before vs. during COVID-19) stratified by gender and age (see Table 4).

**Table 4:** Mental health variables in children and adolescents before vs. during the COVID−19 pandemic, by gender and age.

	**Boy**				**Girl**				**Age 8–13**				**Age 14–17**			
	**Before** **(*n =* 121)**	**During** **(*n =* 41)**			**Before** **(*n =* 168)**	**During** **(*n =* 86)**			**Before** **(*n =* 130)**	**During** **(*n =* 39)**			**Before** **(*n =* 161)**	**During** **(*n =* 90)**		
	**M (SD)**	**M (SD)**	**t_**(160)**_**	***p***	**M (SD)**	**M (SD)**	**t_**(250)**_**	**p**	**M (SD)**	**M (SD)**	**t_**(167)**_**	***p***	**M (SD)**	**M (SD)**	**t_**(249)**_**	***p***
DERS	2.66 (0.73)	3.01 (0.65)	−2.75	0.007	2.97 (0.84)	3.06 (0.81)	−0.82	0.414	2.72 (0.72)	2.78 (0.71)	−0.45	0.650	2.93 (0.86)	3.16 (0.76)	−2.08	0.039
SCARED	30.76 (17.35)	31.68 (15.57)	−0.31	0.756	37.58 (15.91)	35.51 (16.81)	0.96	0.341	32.72 (17.22)	31.59 (15.67)	0.37	0.715	36.21 (16.45)	35.34 (16.72)	0.40	0.691
SMFQ–P	9.56 (7.72)	11.39 (8.04)	−1.34	0.183	13.81 (7.81)	14.71 (8.06)	−1.43	0.150	9.37 (7.39)	10.54 (8.19)	−0.85	0.399	13.47 (7.96)	14.95 (7.86)	−1.36	0.174
SDQ total	16.83 (5.54)	17.62 (5.73)	−80	0.412	16.76 (5.72)	18.62 (6.53)	−2.34	0.020	16.59 (5.50)	18.83 (5.95)	−2.32	0.036	16.95 (5.76)	18.03 (6.42)	−1.38	0.170

Table 4 indicates a significant increase for boys and adolescents in difficulty in emotional regulation (DERS) [t(160) = −2.75; *p* = 0.007] [t(249) = −2.08; *p* = 0.039] during COVID-19. For girls and children there was a small effect increase in SDQ-total [t(250) = −2.34; *p* = 0.020] [t(167) = −2.32; *p* = 0.036] during COVID-19. Z-scores were obtained to examine differences in the prevalence of normal and abnormal reports on the SDQ subscales between COVID-19 samples, stratified by gender and age (see Table 5).

**Table 5:** Prevalence of normal and abnormal reports on SDQ subscales before vs. during the COVID-19 pandemic, stratified by gender and age.

		**SDQ total**	**Emotional**	**Conduct**	**Hyperactivity**	**Social problems**
Before (*n =* 296)						
	Normal	42.6%	37.7%	63.4%	56.1%	57.4%
	Borderline	27.4%	20.2%	12.9%	18.2%	26.7%
	Abnormal	30.1%	42.0%	23.7%	25.7%	15.9%
During (*n =* 132)						
	Normal	31.8%	33.9%	53.8%	52.3%	45.5%
	Borderline	25.8%	14.0%	13.6%	16.7%	28.8%
	Abnormal	42.4%	52.1%	32.6%	31.1%	25.8%
	Z; P	2.49. *p* = 0.006	1.83. *p* = 0.034	1.92. *p* = 0.028	1.15. *p* = 0.124	2.41. *p* = 0.008
Boy (*n =* 171)	Before (*n =* 126)				

Table 5 indicates a significant increase in the prevalence of abnormal reports (SDQ total z = 2.49; *p* = 0.006, Emotional z = 1.83; *p* = 0.034, Conduct z = 1.92; *p* = 0.028, Social Problems z = 2.41; *p* = 0.008) during COVID-19 in all SDQ subscales, except Hyperactivity (z = 1.15; *p* = 0.124). Interestingly, there was a significant increase for girls on the SDQ-total subscale (z = 2.68; *p* = 0.004,), conduct behaviors (z = 2.87; *p* = 0.002,) and social problems (z = 2.05; *p* = 0.020,) subscales, but not for boys. For age groups there was a significant increase in abnormal reports for children (ages 8–13) during COVID-19 on the SDQtotal (z = 2.27; *p* = 0.012), conduct behaviors (z = 2.19; *p* = 0.014,) and social problems (z = 1.97; *p* = 0.025). For adolescents (ages 14–17) there was a significant increase during the pandemic only on the social problems (z = 1.67; *p* = 0.047) subscale.

Finally, a multivariate regression was conducted to examine the effect of difficulty in emotional regulation on all mental health variables (see Table 6).

**Table 6:** DERS impact on SMFQ-C, SDQ-t, SCARED measures stratified by age, gender, and time (before vs. during COVID-19).

**Dependent Variable: SUM_MFQ_C_T**				**Collinearity statistics**
	**B (s.e)**	**Beta**	***P***	**Tolerance**	**VIF**
DERS	7.00 (0.35)	0.69	<0.001	0.94	1.06
Age (1 = 14–17)	1.95 (0.57)	0.19	0.001	0.94	1.07
Gender (1 = girl)	1.73 (0.56)	0.11	0.002	0.95	1.06
Time (1 = during)	0.12 (0.59)	0.01	0.836	0.97	1.03
F	125.89; *p* < 0.001				
R^2^	0.56				
**Dependent Variable: SUM_SCAED_C**	**B (s.e)**	**Beta**	***P***	**Tolerance**	**VIF**
DERS	13.72 (0.81)	0.65	<0.001	0.94	1.06
Age (1= 1 4–17)	−0.44 (1.33)	−0.01	0.741	0.94	1.07
Gender (1 = girl)	2.84 (1.32)	0.08	0.031	0.95	1.06
Time (1=during)	−2.74 (1.39)	−0.08	0.045	0.97	1.03
F	78.13; *p* < 0.001				
R^2^	0.44				
**Dependent Variable: SDQ_T_C**	**B (s.e)**	**Beta**	***P***	**Tolerance**	**VIF**
DERS	4.10 (0.31)	0.56	<0.001	0.94	1.06
Age (1 = 14–17)	−0.98 (0.51)	−0.08	0.054	0.94	1.07
Gender (1 = girl)	−0.54 (0.51)	−0.05	0.287	0.95	1.06
Time (1 = during)	1.22 (0.53)	0.10	0.023	0.97	1.03
F	45.70; *p* < 0.001				
R^2^	0.32				

Table 6 indicates that difficulty in emotional regulation predicts all mental health variables, suggesting that greater difficulty in emotional regulation (DERS) (B = 7.03; SE = 0.35) predict higher depressed moods (SMFQ-C), anxiety (SCARED) and emotional and behavioral problems (SDQ-t). When examining these predications stratified by age, gender and time, difficulty in emotional regulation predicts significantly higher depressed moods and lower emotional and behavioral problems in adolescents (B = 1.93; SE = 0.76) and higher depressed moods and anxiety in girls (B = 2.81; SE = 1.31) (B = 1.71; SE = 0.57) with an explained variance of 56%. Greater difficulties in emotion regulation predicts higher anxiety before COVID-19 (B = −2.81; SE = 1.38) and increased emotional and behavioral difficulties during the pandemic, with an explained variance of 32%.

## Discussion

While there is a growing amount of scientific literature to support the notion that children and adolescents are, overall, a population at risk for mental health difficulties (58), and more specifically during the COVID-19 pandemic, some studies have found a differential impact among certain sub-groups (10, 59, 60). Few of these studies examined clinical samples of children and adolescents, suggesting a range of factors that need to be considered to understand the effects on this vulnerable population [e.g., (29, 61)]. Along these lines, the aim of our study was to examine the experience of burden by COVID-19 and difference in mental health symptoms before vs. during the pandemic in a clinical sample of children and adolescents who were referred to a public mental health clinic. Our findings indicated that most children and adolescents (82%) from the “during pandemic” sample reported that their feelings worsened during the pandemic. As in other studies, girls reported more burden than boys and adolescents more than children (19, 22). In the current study, this reported burden was associated with anxiety, emotional and behavioral problems, and difficulty in emotional regulation, suggesting that feeling worse is associated with higher mental health symptoms excluding depressed mood. For girls and adolescents, these associations were significant, and for boys feeling worse was significantly associated only with anxiety. These findings resemble both Brailovskaia et al. (4, 5) surveys of adults in Europe, and Ravens-Sieberer et al. (10) findings that studied children and adolescents. However, we studied a clinical sample of children and adolescents, and therefore the indication that burden by COVID-19 was not associated with depressed moods and specifications of age and gender are important for clinical implications in this subgroup.

When comparing the before vs. during pandemic samples, our findings aligned with other studies that indicated an increase in children's and adolescents' depressed moods (28) and emotional and behavioral problems during the pandemic (24). In addition, we found an increase in difficulty in emotional regulation during pandemic, suggesting that the overall stress experienced by families in the beginning of the pandemic coupled with the shutdown of all life routines affected participants emotional regulation. This increase may reflect the importance and necessity of school and other daily routines as external support to developing inner processes of emotional regulation in children and youth (44). In the absence or change of these routines it was difficult for the not-yet fully developed emotional regulation ability to deal with the increasing negative feelings following COVID-19 (29).

Interestingly and contrary to most other studies, our findings did not indicate an increase in anxiety during the pandemic. This may be because most studies examined children and adolescents of the general population (17) while we studied a clinical sample characterized by high anxiety prior to the pandemic, and therefore no significant difference was detected between the two different time periods (before vs. during the pandemic). Stewart et al. (60) found similar findings in a clinical sample and suggested that perhaps participants who previously received one or more forms of mental health support already have some necessary tools that result in improved coping to manage changes and stress brought on by the COVID-19 pandemic (60). We did not collect any information regarding mental health support and therefore our interpretation in this direction is limited. When comparing samples stratified by gender and age, our findings indicated a significant difference in difficulty in emotional regulation and emotional and behavioral problems. Boys and adolescents reported a significant increase in difficulty in emotional regulation and girls and children reported an increase in emotional and behavioral problems. While the increase in emotional and behavioral problems aligns with other studies [e.g., (19, 62)] the more challenging finding is the increase in difficulty in emotional regulation. Since a dearth of studies have examined difficulty in emotional regulation during the pandemic, comparison is problematic, but from studies that found gender differences in mental health symptoms during COVID-19 (12, 25, 26), we assumed that the disruption in the daily routine and the social isolation affected boys' and adolescents' emotional regulation more than girls' and childrens (47). Previous studies found that females employ more adaptive (e.g., active coping and re-evaluation) emotion regulatory strategies than males (63). Females were also found to better identify, label and express feelings, to be more aware of their emotions and more open to engaging with their emotions (64). When examining the prevalence of normal and abnormal reports on SDQ subscales before vs. during COVID-19 pandemic, we found that in all subscales excluding Hyperactivity, children and adolescents reported a significant higher prevalence of abnormal reports. For girls and children, the increase was significant in the total problems, conduct and peer relationship problems, and for adolescents there was a significant increase only in the peer relationship problem. These findings may reflect the accumulated evidence about the clinical change in the referred population to public mental health services, suggesting that before the pandemic most girls were referred to the service due to high levels of anxiety while during the pandemic their referral was due to eating disorders (15). Also, it seems that children are more vulnerable to emotional and behavioral problems during quarantine, as they have fewer psychological resources for coping with stressful circumstances in comparison to youth, fewer opportunities to maintain contact with other people during quarantine using electronic devices (65), and rely almost exclusively on their caregivers as relational sources (66).

Finally, we found that greater difficulty in emotional regulation predicted increased depressed moods, anxiety, and emotional and behavioral problems for the entire sample. Depressed moods were predicted significantly higher in adolescents and girls, while emotional and behavioral problems were predicted significantly higher in children during the pandemic, and higher anxiety in girls before COVID-19. These results strongly follow the emotion regulation perspective, suggesting that a difficulty to regulate negative emotions is a central feature of depression and anxiety, and more so in females that tend to internalize negative feelings (40). Whereas males and younger children tend to project negative emotions, feel anger, and express the dysregulation of emotion in behavioral problems (44). It may also suggest that for younger children the inflexible use of emotional regulation strategies or a deficit in this ability is expressed in externalizing symptoms (67).

While our findings support the critical role of difficulty in emotional regulation for children and adolescents' mental health symptoms, the effects of these difficulties during the pandemic were indicated only on emotional and behavioral problems. Other studies that examined difficulty in emotional regulation in adults during the pandemic suggested that difficulty in emotion regulation is associated with poorer mental health symptoms during crises and chronic stressors, including the COVID-19 pandemic (35, 36). These findings have important clinical implications as they suggest that improving emotion regulation abilities could be a protective factor, particularly for at-risk adolescents, and for coping with chronic stressors such as the COVID-19 pandemic. However, further research is needed to explore the possible transdiagnostic mechanism of difficulty in emotional regulation on mental health symptoms in children and youth.

Despite these interesting findings, some limitations of this study should be considered. This study's population consisted of children who arrived at a community mental health clinic; therefore, their report regarding the intensity of their emotional and behavioral difficulties may have been biased. The sample size was good but the “during pandemic” sample was limited to those children and families that decided to seek help during the pandemic in a public mental health service. Furthermore, this was a cross-sectional study based on self-reporting tools; thus, the research limitations that characterize this type of research and the limitations of social desirability should be considered. Finally, it should be noted that the population sample was from a peripheral area of Israel and may not be representative of the general population.

## Conclusions

Overall, our findings highlight the significant increase in mental health symptoms of children and adolescents during the COVID-19 pandemic. They also provide evidence for gender and age differences that need to be further explored and taken in consideration. Specifically, they reflect the vulnerability of this young population, and the importance of prevention measures and clinical practice to provide suitable support in the present crisis and comparable future situations. In addition, our findings contribute to the accumulating knowledge regarding the effect of difficulty in emotional regulation on mental health symptoms in children and adolescents, and the need to further explore this variable as a transdiagnostic variable. Since COVID-19 is an ongoing situation with multiple and long-term effects, the mental health of children and adolescents needs to be carefully monitored to assess and prevent long-term impact and to investigate resources and resilience factors which may benefit children's coping abilities.

## Data Availability Statement

The raw data supporting the conclusions of this article will be made available by the authors, without undue reservation.

## Ethics Statement

The studies involving human participants were reviewed and approved by Ziv Medical Center Ethics Committee. Written informed consent to participate in this study was provided by the participants' legal guardian/next of kin.

## Author Contributions

All authors contributed equally to planning the study, collection of data, data analysis, and writing this paper.

## Conflict of Interest

The authors declare that the research was conducted in the absence of any commercial or financial relationships that could be construed as a potential conflict of interest.

## Publisher's Note

All claims expressed in this article are solely those of the authors and do not necessarily represent those of their affiliated organizations, or those of the publisher, the editors and the reviewers. Any product that may be evaluated in this article, or claim that may be made by its manufacturer, is not guaranteed or endorsed by the publisher.
